# The potential of the Crystal Cam handheld gamma-camera for preoperative and intraoperative sentinel lymph node localization in early-stage oral cancer

**DOI:** 10.1007/s00405-023-08138-y

**Published:** 2023-07-26

**Authors:** Rutger Mahieu, Bernard M. Tijink, Robert J. J. van Es, Bastiaan J. van Nierop, Casper Beijst, Bart de Keizer, Remco de Bree

**Affiliations:** 1 Department of Head and Neck Surgical Oncology, University Medical Center Utrecht, Heidelberglaan 100, 3584 CX Utrecht, The Netherlands; 2 Department of Radiology and Nuclear Medicine, University Medical Center Utrecht, Utrecht, The Netherlands

**Keywords:** Oral cancer, Sentinel lymph node biopsy, Lymphatic metastasis, Lymphoscintigraphy, SPECT, CT, Gamma-camera

## Abstract

**Purpose:**

Evaluating the Crystal Cam handheld gamma-camera for preoperative and intraoperative sentinel lymph node (SLN) localization in early-stage oral cancer.

**Methods:**

The handheld gamma-camera was used complementary to conventional gamma-probe guidance for intraoperative SLN localization in 53 early-stage oral cancer patients undergoing SLN biopsy. In 36 of these patients, a blinded comparison was made between preoperative handheld gamma-camera and lymphoscintigraphy outcomes. Of those, the reliability for marking the SLN’s location using both handheld gamma-camera and a ^57^Co-penpoint marker was evaluated in 15 patients.

**Results:**

In the entire cohort, the handheld gamma-camera preoperatively detected 116/122 (95%) of SLNs identified by lymphoscintigraphy. In those patients where the observer was blinded for lymphoscintigraphy (*n* = 36), 71/77 (92%) SLNs were correctly identified by handheld gamma-camera. Overlooked SLNs by handheld gamma-camera were mainly located near the injection site. The SLN’s marked location by handheld gamma-camera and ^57^Co-penpoint marker was considered accurate in 42/43 (98%) SLNs. The intraoperative use of the handheld gamma-camera led to the extirpation of 16 additional ‘hot’ lymph nodes in 14 patients, 4 of which harbored metastases, and prevented 2 patients (4%) from being erroneously staged negative for nodal metastasis. In those with follow-up ≥ 24 months or false-negative outcomes < 24 months following SLNB, a sensitivity of 82% and negative predictive value of 93% was obtained.

**Conclusion:**

The Crystal Cam handheld gamma-camera offers reliable preoperative and intraoperative SLN localization and might reduce the risk of missing a malignant SLN during surgery. Detecting SLNs near the injection site by handheld gamma-camera remains challenging.

## Introduction

Over the last decade, sentinel lymph node biopsy (SLNB) is being increasingly advocated for staging the clinically negative neck (cN0) in early-stage oral squamous cell carcinoma (OSCC) [1, 2].

Recent trials have confirmed the less-invasive character of SLNB in OSCC, with lower functional morbidity and similar oncological outcomes when compared to elective neck dissection (END) [3–5].

However, as the rate of false-negative SLNB still varies between 5 and 15%, with its accompanying oncological implications (i.e., comprehensive surgery, adjuvant radiotherapy and reduced disease-specific survival), efforts are made to further improve the accuracy of SLNB, especially in floor-of-mouth cancers [5–10].

Several novel lymphographic techniques have been proposed to improve preoperative identification of sentinel lymph nodes (SLN) [9]. However, tracking the preoperatively identified SLNs during surgery can remain a challenge.

Conventionally, SLNs identified by lymphoscintigraphy (including SPECT/CT) are localized intraoperatively through gamma-tracing using a handheld gamma-probe [11]. Handheld gamma-probes have some limitations as their performance is operator-dependent, lack the ability for visual feedback and provide inadequate contrast for differentiating between neighboring radioactive signals [11, 12]. Especially in cases where SLNs are identified close to the injection site, distinction between SLN and injection site by gamma-probe can be complicated [10–13].

Optical tracers have been suggested, such as various blue dyes and indocyanine green (ICG), but these pose several limitations as well. Since unbound optical tracers appear to flow quickly to SLNs, yet are not retained in lymph nodes, they may washout or migrate to higher echelon nodes (HEN) by the time of SLN retrieval [10]. The adjunction of fluorescent dyes to well-known radiotracers (e.g., ICG-^99m^Tc-nanocolloid), on the other hand, has shown promising results for intraoperative SLN localization [14–16]. Nevertheless, radioguidance remains the cornerstone of SLN localization, owing to the limited tissue penetration of the fluorescent signal (0.5–1 cm), hampering the use of fluorescent dyes for surgical planning or for tracking SLNs from larger distances [10, 15].

Various portable gamma-detecting imaging devices have been developed for visualization of radiotracers (e.g., freehand SPECT, portable gamma-cameras), allowing for real-time image-guided SLN localization while being less affected by tissue attenuation compared to fluorescence guidance. The complementary use of real-time radioguided imaging to standard handheld gamma-probe guidance has shown to facilitate more accurate and efficient localization of SLNs during surgery [12, 13, 17–21].

However, most portable gamma-cameras and freehand SPECT devices are large in size, costly and occasionally require an additional operator to establish optimal settings. The handheld gamma-camera used in this study (Crystal Cam, Crystal Photonics GmbH, Berlin, Germany) is relatively inexpensive and highly portable, owing to its small size and low weight (see ‘*Specifications Crystal Cam*’), which can be fully managed by the surgeon in a sterile setting. Previously, the feasibility and utility of this handheld gamma-camera for SLN localization has been described in melanoma and breast cancer patients [22–25].

In this prospective study, the utilization of this handheld gamma-camera is evaluated for preoperative and intraoperative SLN localization in early-stage OSCC patients undergoing SLNB.

## Materials and methods

### Patients

This study was performed in line with the principles of the Declaration of Helsinki and was approved by the University Medical Center Utrecht’s Ethics Committee (no. 17/835); informed consent for participation was obtained from all the patients.

Between January 2018 and October 2020, a total of 53 patients with clinically T1-T3N0 OSCC scheduled for SLNB were prospectively included (Table 1; TNM Staging AJCC UICC 8th Edition [26, 27]). Patients with a tumor clinically staged as T3 were only included when staging was based on depth-of-invasion of > 10 mm and tumor dimensions of > 2 cm and ≤ 4 cm [28].

**Table 1:** Patient- and tumor characteristics

Characteristics	*n* = 53
Gender, *n* (%)	
Female	24 (45%)
Median age (y) (range)	61.5 (29–97)
History of head and neck cancer, *n* (%)	10 (19%)
Previous oncological treatment of the neck, *n* (%)	
Neck dissection	6 (11%)
Radiotherapy	1 (2%)
Neck dissection and chemoradiation	1 (2%)
Tumor location, *n* (%)	
Tongue	31 (59%)
Floor-of-mouth	5 (9%)
Buccal mucosa	7 (13%)
Retromolar trigone	7 (13%)
Lower gum	3 (6%)
Side primary tumor, *n* (%)	
Left	21 (40%)
Right	30 (56%)
Midline	2 (4%)
Radiotracer, *n* (%)	
Nanocolloid	39 (74%)
Tilmanocept	14 (26%)
Two-day SLNB protocol, *n* (%)	47 (89%)
Pathological T-stage, *n* (%)^a^	
pT1	19 (36%)
pT2	29 (55%)
pT3	4 (7%)
pT4a	1 (2%)
Median harvested SLNs (range)	3 (0–6)
Histopathological status SLNs, *n* (%)	
Negative	123 (89%)
Positive	15 (11%)
SLNB outcome, *n* (%)	
pN0(sn)	43 (81%)
pN+(sn)	10 (19%)
Complementary neck treatment, *n* (%)	
Neck dissection	5 (9%)
Radiotherapy	5 (9%)
Pathological N-stage, *n* (%)^a^	
pN1	3 (6%)
pN2b	5 (9%)
pN2c	1 (2%)
pN3b	1 (2%)
Follow-up in months (range)	23 (2–49)

*n* number, *y* years, *mm* millimeters, *SD* standard deviation, *SLNs* sentinel lymph nodes, *SLNB* sentinel lymph node biopsy, *ND* neck dissection; RT, radiotherapy

^a^According to AJCC TNM classification, 8th edition

In all patients, cN0 status was determined by ultrasound of the neck. In those with suspect lymph nodes, ultrasound-guided fine-needle aspiration cytology was performed. The majority of patients (72%) also underwent magnetic resonance imaging of the head and neck as part of their clinical staging.

### Study design

**Fig. 1:**
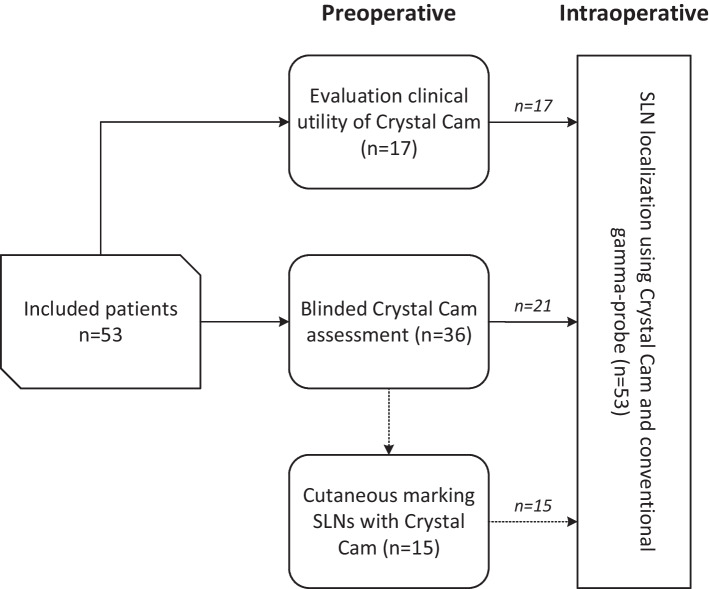
Flowchart of study procedures. Dotted arrow represents patients (*n* = 15) who underwent both blinded assessment as well as cutaneous marking of the SLNs’ location by handheld gamma-camera and a ^57^Co-penpoint marker. *n* number, *SLN* sentinel lymph node

This study was performed in several phases. Figure 1 provides an overview of the study procedures and included patients for each phase of the study.

**Fig. 2:**
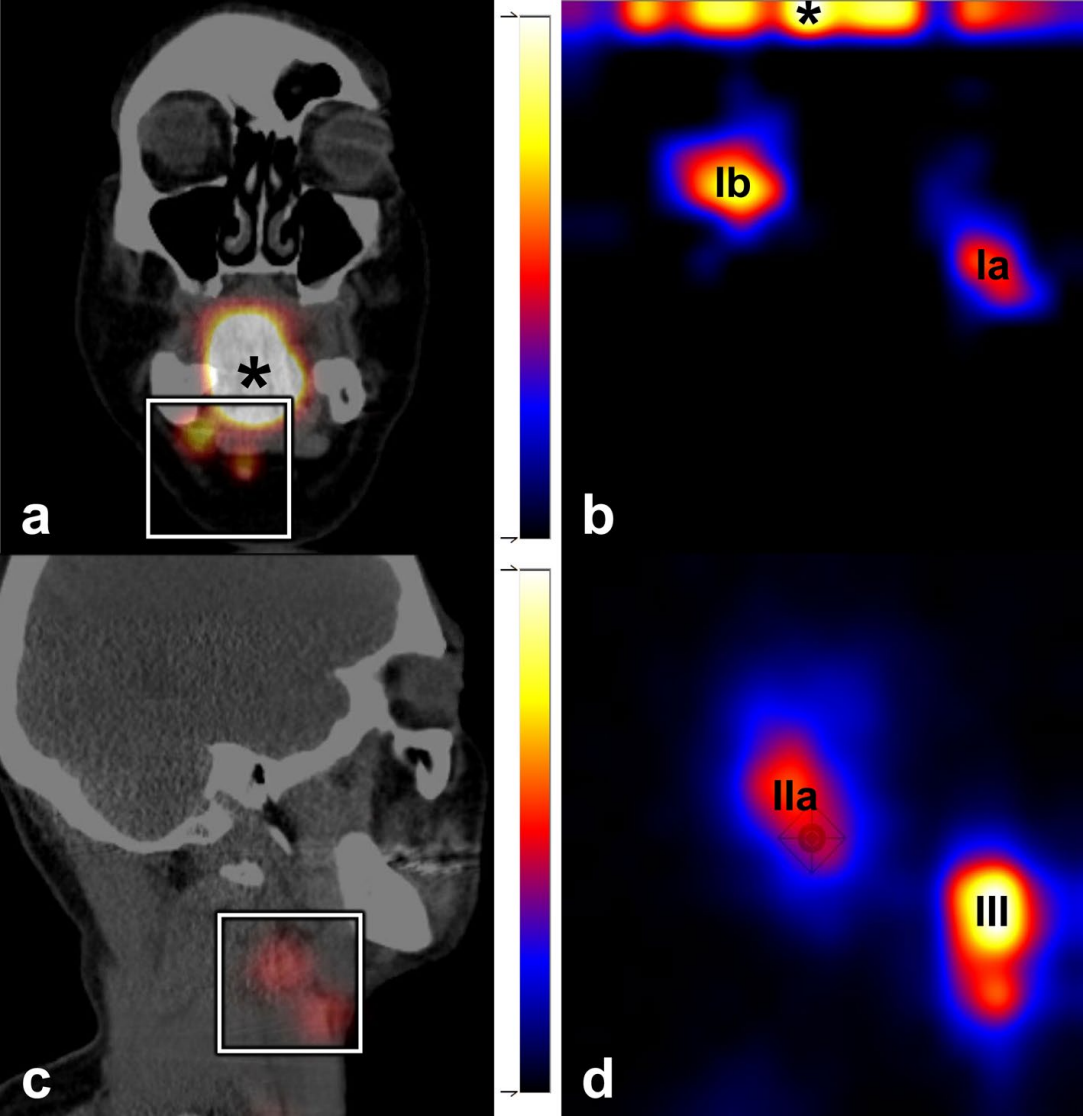
Comparison SPECT/CT (**a**, **c**) and Crystal Cam handheld gamma-camera images (**b, d**). Coronal plane of SPECT/CT (**a**) depicting injection site (*) and two SLNs located in level Ia and level Ib on the left side. Both SLNs (*Ia, Ib*) and injection site could be visualized within the field-of-view of the handheld gamma-camera (white square, **a**) (**b**). Two SLNs as identified by SPECT/CT (**c**) (sagittal plane; level IIa, III), also detected by handheld gamma-camera (*IIa, III*) (**d**)

First, the clinical utility of this handheld gamma-camera was evaluated in 17 patients (32%), by assessing whether identified SLNs using lymphoscintigraphy could be detected preoperatively with the handheld gamma-camera (Fig. 2).

Subsequently, to evaluate the reliability of SLN identification using the handheld gamma-camera, a blinded comparison was made between preoperative handheld gamma-camera and lymphoscintigraphy outcomes in 36 patients (68%; see ‘Assessment blinded for lymphoscintigraphy’).

Out of those who underwent blinded SLN assessment by handheld gamma-camera (n = 36), the reliability of SLN localization using the handheld gamma-camera and a ^57^Co-penpoint marker was evaluated in 15 patients (42%; see ‘Cutaneous marking location SLNs’).

In all patients (*n* = 53), the handheld gamma-camera was used complementary to conventional gamma-probe guidance for intraoperative SLN localization (see ‘Surgical procedure’).

### Specifications

**Fig. 3:**
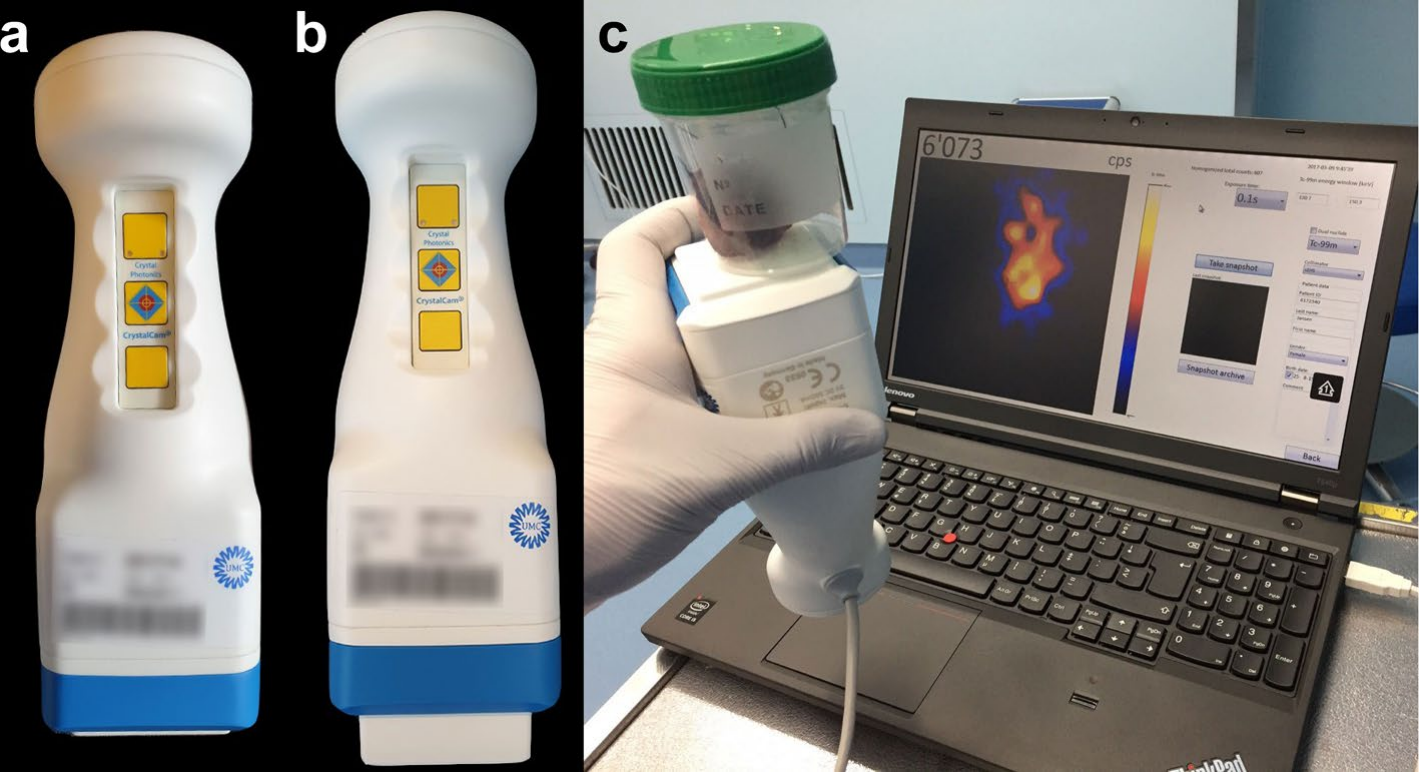
Crystal Cam handheld gamma-camera. **a** Equipped with a low-energy high-sensitivity (LEHS) collimator. **b** Equipped with a low-energy high-resolution (LEHR) collimator. **c** Connected to laptop with Crystal Imager software via USB

The Crystal Cam is a handheld solid-state gamma-camera with a cadmium zinc telluride detector (thickness: 5 mm), that provides two-dimensional imaging at a field-of-view of 40 × 40 mm^2^ with 16 × 16 pixels (Fig. 3). Its physical dimensions (65 × 65 × 180 mm) and total weight of 0.8 kg, including a collimator and 3 mm lead integrated side shielding, allow for single-handed control of the gamma-camera without an articulated arm. The included low-energy (LE) collimators, which can be changed at runtime, facilitate either high sensitivity (LEHS collimator; Fig. 3a) or high-resolution (LEHR collimator; Fig. 3b) imaging. Both the LEHS and LEHR collimator were used at discretion of the observers. Control and visualization software (Crystal Imager) runs on a standard laptop to which the handheld gamma-camera is connected (Fig. 3c). This handheld gamma-camera is able to simultaneously detect both ^99m^Tc and ^57^Co in different energy windows [23].

In this study, quality controls of the handheld gamma-camera were performed on a regular basis by a nuclear physicist for testing sensitivity, homogeneity, peaking and null-effect.

### Lymphoscintigraphy

Lymphoscintigraphy including SPECT/CT acquisition were conducted according to EANM guidelines [11]. Following peritumoral injections of a ^99m^Tc-labeled radiotracer (i.e., ^99m^Tc-nanocolloid or ^99m^Tc-tilmanocept) planar static and dynamic scintigraphy as well as SPECT/CT were acquired the day prior to surgery (2-day protocol) or the day of surgery (single-day protocol) on a Siemens Symbia T16 SPECT/CT scanner; equipped with low-medium energy (LME) collimators to limit septal penetration and reduce shine-through [29]. SPECT images were reconstructed using clinical reconstruction software (Siemens Flash3D), with attenuation and scatter correction (6 iterations, 8 subsets, 5 mm Gaussian filter). For the 2-day protocol, ~ 120 MBq ^99m^Tc-nanocolloid or ~ 74 MBq ^99m^Tc-tilmanocept was administered, whereas for the single-day protocol, ~ 50 MBq ^99m^Tc-nanocolloid was administered.

### Assessment blinded for lymphoscintigraphy

Immediately following lymphoscintigraphy, SLN assessment was preoperatively performed using the handheld gamma-camera by a single observer while blinded for lymphoscintigraphy (*n* = 36; 68%). Identified hotspots using the handheld gamma-camera were recorded and designated as either SLN or HEN on the basis of their location and relative radioactive intensity. Subsequently, the results of lymphoscintigraphy including SPECT/CT, as reviewed by a nuclear physician, were revealed to the blinded observer. Any discrepancies between lymphoscintigraphy and handheld gamma-camera outcomes were registered. If SLNs were missed by blinded assessment using the handheld gamma-camera, an additional assessment was conducted to determine whether missed SLNs could be identified with either the handheld gamma-camera or gamma-probe with information provided by lymphoscintigraphy. In all patients, lymphoscintigraphy was leading in designating SLNs for biopsy.

### Cutaneous marking location SLNs

**Fig. 4:**
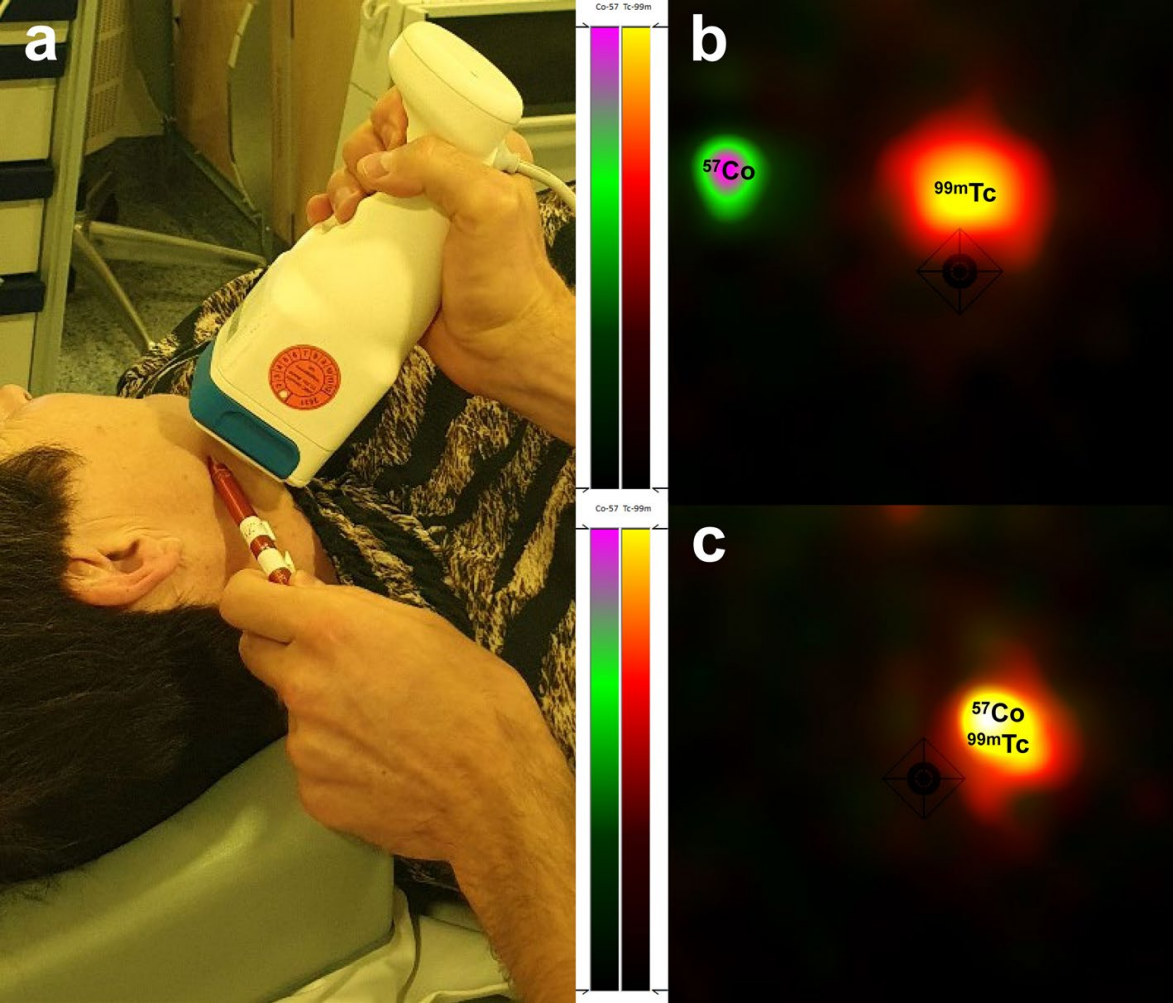
**a** Cutaneous marking of the location of SLNs using Crystal Cam handheld gamma-camera and a ^57^Co-penpoint marker. To simulate the definite surgical position, the patient is placed in supine position with head slightly extended and rotated to the opposite side. **b** Separate ^57^Co-hotspot (^*57*^*Co*) and ^99m^Tc-hotspot (^*99m*^*Tc*) within the handheld gamma-camera’s field-of-view. (**c**) Overlapping ^57^Co-hotspot and ^99m^Tc-hotspot, indicating that the ^57^Co-penpoint marker is positioned on the SLN’s location

In 15 patients (28%), the location of the SLNs designated for biopsy by lymphoscintigraphy were first marked on the overlying skin with the handheld gamma-camera and a ^57^Co-penpoint marker using its dual-isotope function (Fig. 4). Then, with the patient in a similar position, the location of the identified SLNs were marked using the conventional gamma-camera (Siemens Symbia T16 system) and the ^57^Co-penpoint marker, according to standard protocol. Subsequently, the location of both cutaneous markings were compared, with cutaneous markings based on the conventional gamma-camera as reference standard. The marked location of SLNs using the handheld gamma-camera was considered accurate if they deviated ≤ 10 mm in any direction from the location as marked with the conventional gamma-camera.

### Surgical procedure

**Fig. 5:**
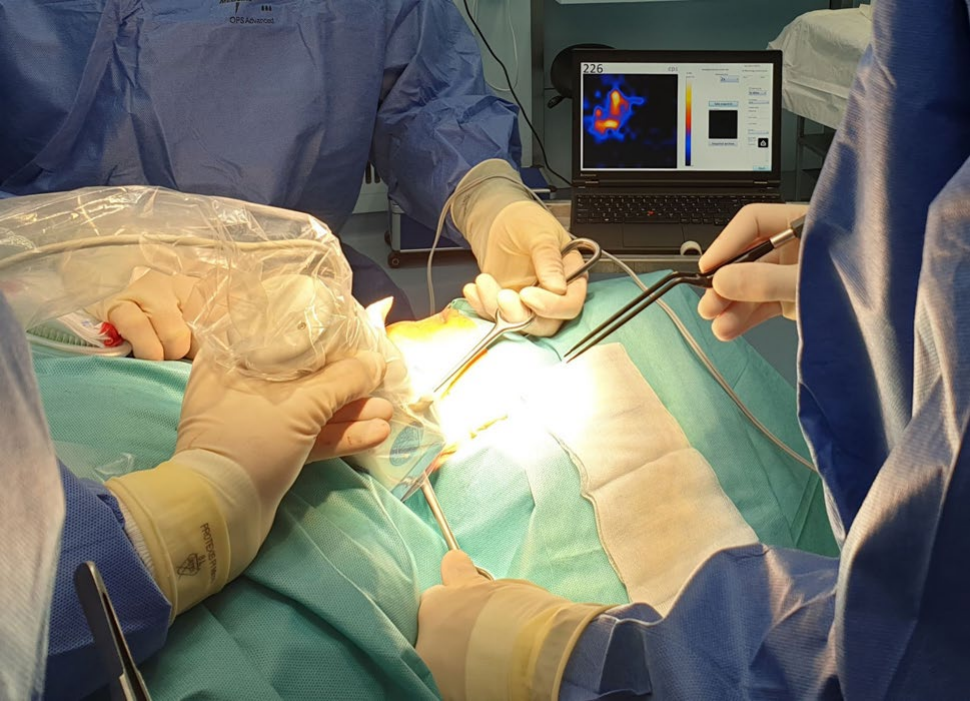
Intraoperative use of Crystal Cam handheld gamma-camera

Marked SLNs were localized and harvested primarily under conventional portable gamma-probe guidance; the handheld gamma-camera was available to the surgeon on request. During surgery, an experienced operator was present to assist in using the handheld gamma-camera. In 10 patients (19%), also fluorescence guidance was available using ICG-^99m^Tc-nanocolloid and near-infrared imaging. The location of harvested SLNs including their radioactive uptake (in counts per second as measured by the portable gamma-probe) were registered. Following extirpation of a SLN and check for residual activity with the gamma-probe, the handheld gamma-camera was used to scan for residual activity (Fig. 5).

At the end of SLNB, surgeons were asked whether the handheld gamma-camera further assisted SLN localization when used complementary to conventional portable gamma-probe guidance. To this end, the 3-point subjective scoring system as previously published by Heuveling et al. was adopted [12]:

(1) the handheld gamma-camera provided confusing information and was not helpful; (2) the handheld gamma-camera could reliably be used, but provided no additional helpful information; (3) the handheld gamma-camera provided additional helpful information for localization of SLNs.

### Histopathology and follow-up

Harvested SLNs were sent for histopathological examination using step-serial-sectioning (section thickness 150 μm) with hematoxylin–eosin and pan-cytokeratin antibody (AE 1/3) staining [30, 31]. Patients with histopathologically negative SLNs were assigned to a wait-and-scan approach. In those with at least one histopathologically positive SLN, complementary treatment of the affected and adjacent nodal basins was employed (i.e., neck dissection and/or (chemo)radiotherapy). Complementary neck dissection specimens were histopathologically assessed for additional (non-SLN) nodal metastases. Follow-up visits were scheduled according to standard oncological care.

### False-negative SLNB

Isolated regional recurrences that occurred in the side of the neck staged negative by SLNB, within 3 years following treatment, were regarded as a false-negative outcome for SLNB. Regional recurrences in the presence of local recurrence or second primary tumors were not considered false-negative outcomes for SLNB, as differentiation between missed nodal metastasis at initial diagnostic work-up and metastasis developed from a reseeding local recurrence or second primary tumor is unfeasible.

### Analyses

All data were analyzed with IBM SPSS Statistics Version 28.0 (IBM Corp., Armonk, New York, United States). Descriptive statistics are presented as number of cases and percentages for dichotomous and ordinal variables, whereas continuous parametric variables are presented as mean and standard deviation (SD). Non-parametric variables are presented as median with interquartile range (IQR). Fisher’s exact test was used to compare categorical variables containing small number of cases (*n* ≤ 5).

Spearman’s rank-order correlation tests were conducted to determine the association between amount and location of identified SLNs by blinded handheld gamma-camera assessment and lymphoscintigraphy for each patient.

On the basis of the false-negative rate for SLNB in this cohort, the sensitivity (true positives/(true positives + false negatives)) and negative predictive value (NPV; (true negatives/(true negatives + false negatives)) were calculated.

Overall, a *p*-value ≤ 0.05 was considered to be statistically significant.

## Results

Patient and tumor characteristics of included patients are listed in Table 1. A total of 10 (19%) patients had a history of head and neck cancer, of which 8 previously underwent oncological treatment of the neck. In the majority of patients, the primary tumor was located in the oral tongue (59%). Most tumors were pathologically classified as pT2 (55%). In one patient, the primary tumor (retromolar trigone) was pathologically classified as pT4a on the basis of mandibular invasion.

Patients mainly underwent SLNB by 2-day protocol (47/53; 89%) using ^99m^Tc-nanocolloid (33/47; 70%). A total of 138 SLNs were harvested, on average 3 per patient (range 0–6), of which 15 harbored metastasis (11%). Of those patients with nodal metastases as assessed by SLNB (*n* = 10; 19%), half underwent complementary neck dissection whereas the other half underwent complementary radiotherapy. Two patients underwent adjuvant neck irradiation following complementary neck dissection. None underwent concurrent chemotherapy as part of complementary- or adjuvant therapy.

### Identification of SLNs

In the 17 patients in whom the clinical utility of the handheld gamma-camera was evaluated, 41 out of the 45 SLNs (91%) identified by lymphoscintigraphy were also detected with the handheld gamma-camera. The undetected SLNs (*n* = 4) in two patients were located in levels Ia, IIa, III and V of the ipsilateral neck (Table 2). None of these undetected SLNs could be localized intraoperatively as its radioactive signal was either indistinguishable from the radioactive signal deriving from the injection site or on account of their marginal radioactive uptake. In both the patients, there was no evidence of nodal disease at 31- and 35-month follow-up.

**Table 2:** Undetected and/or overlooked SLNs by Crystal Cam handheld gamma-camera

Primary tumor	Previous neck treatment	Radiotracer (*dosage*)	Identified SLNs LSG		Harvested	PA	pN(sn)^a^	Complementary treatment	Follow-up (months*)*
Buccal mucosa (*left*)	SND left	Nanocolloid (*126 MBq*)	IIa	Right	Yes	–	pN0	None	NED (35)
			V	Left*	No	N.A			
Tongue (*right*)	None	Tilmanocept (*74 MBq)*	IIa	Right*	No	N.A	pN2c	Bilateral ND	NED (31)
			III	Right*	No	N.A			
			Ia	Left*	No	N.A			
			Ib	Left	Yes	+			
			III	Left	Yes	–			
Lower gum (*right*)	None	Nanocolloid (*117 MBq*)	Ia	Right*^,b^	Yes	–	pN0	None	NED (37)
			Ib	Right*^,b^	Yes	–			
			IIa	Right	Yes	–			
			III	Right	Yes	–			
Buccal mucosa (*left*)	None	Nanocolloid (*122 MBq*)	Ib	Left	Yes	+	pN1	Unilateral RT	NED (30)
			Ib	Left*^,b^	No	N.A			
Tongue (*right*)	None	Nanocolloid (*131 MBq*)	Ib	Right^b^	Yes	–	pN0	None	NED (29)
			III	Right	Yes	–			
			IIa	Left	Yes	–			
			III	Left	Yes	–			
Tongue (*right*)	SND right	Nanocolloid (*119 MBq*)	Ia	Left	Yes	+	pN0	None	NED (26)
			IIa	Right*^,b^	No	N.A			
Tongue (*right*)	None	Nanocolloid (*60 MBq*)	IIa	Right	Yes	–	pN0	None	NED (20)
			IIa	Left	Yes	–			
			III	Left^b^	Yes	–			

*SLN* sentinel lymph node, *LSG* lymphoscintigraphy, *PA* pathological assessment, *SND* selective neck dissection, *MBq* megabecquerel, *N.A.* not applicable, +  histopathologically positive for metastasis, – histopathologically negative for metastasis, *NED* no evidence of disease, *ND* neck dissection, *RT* radiotherapy

*Undetected by handheld gamma-camera

^a^According to AJCC TNM classification, 8th edition

^b^Overlooked by blinded handheld gamma-camera assessment

While blinded for lymphoscintigraphy (*n* = 36), 71 out of 77 SLNs were correctly identified using the handheld gamma-camera (92%). The SLNs as overlooked by blinded handheld gamma-camera assessment were mainly located close to the injection site (levels Ia and Ib); one was located in the previously treated neck and one was located in the contralateral neck of the primary tumor (Table 2). Of the 6 overlooked SLNs by blinded assessment in 5 patients, only 4 were surgically harvested since the other two could not be located intraoperatively by neither handheld gamma-probe nor handheld gamma-camera. Histopathological assessment showed no metastasis in these 4 overlooked SLNs. The two patients in whom the overlooked SLNs could not be harvested intraoperatively showed no evidence of nodal disease 26 and 30 months following SLNB.

In 4 patients, a total of 6 hotspots were scored as SLN based on blinded handheld gamma-camera assessment, but designated as HEN based on lymphoscintigraphy. In addition, 2 SLNs were incorrectly identified with the handheld gamma-camera (false-positives): one revealed to be an appendix of the injection site whereas the other appeared to be an intense hotspot in the contralateral neck, which was mistaken for a SLN in the side of the neck being scanned.

Overall, there was a strong agreement between SLNs identified using the handheld gamma-camera while blinded for lymphoscintigraphy and the SLNs as ultimately designated for biopsy by lymphoscintigraphy (*r*_s_ = 0.857, *p* < 0.001).

### Cutaneous marking location SLNs

In 15 patients, the location of 43 SLNs designated for biopsy was marked on the overlying skin with the handheld gamma-camera and the ^57^Co-penpoint marker. The marked location by handheld gamma-camera deviated on one occasion 15 mm from the location as marked by the conventional gamma-camera and for all other instances on average 1.0 mm (range 0–10 mm).

### Intraoperative SLN localization

Intraoperatively, based on the information as provided by the complementary use of the handheld gamma-camera, 16 additional ‘hot’ lymph nodes were harvested in 14 patients (26%). Out of these additionally harvested lymph nodes, 4 harbored metastases as confirmed by histopathological assessment (25%) which led to upstaging in 4 patients (8%). On account of these additionally harvested metastatic lymph nodes, two patients underwent complementary treatment of the neck instead of being assigned to a wait-and-scan approach [pN0(sn) to pN1(sn)] and one patient was upstaged from pN1(sn) to pN2b(sn), which had no therapeutic consequences since the patient chose to undergo complementary radiotherapy instead of a complementary neck dissection followed by adjuvant neck irradiation. The remaining patient was upstaged from pN1(sn) to pN3b(sn), yet opted for complementary radiotherapy only and declined a complementary neck dissection or concurrent chemotherapy.

In 29 patients (55%), the surgeon deemed the complementary use of the handheld gamma-camera as “helpful” for localizing SLNs intraoperatively. In the remaining patients (*n* = 24), the performance of the handheld gamma-camera was considered reliable, but did not provide additional helpful information (45%). None regarded the information provided by the handheld gamma-camera as “confusing”.

For the 10 patients in whom also fluorescence guidance was available, the handheld gamma-camera was still considered to be of added value in 4 patients (surgeon score 3; 40%), which did not differ significantly when compared to the rate of patients in whom no fluorescence guidance was available and the handheld gamma-camera was regarded as helpful (58%; *p* = 0.482). In these 10 patients, 2 additional ‘hot’ lymph nodes were harvested on account of information provided by the handheld gamma-camera, none of which harbored metastasis.

### Follow-up

In this cohort, two patients developed an isolated nodal recurrence in the side of the neck staged negative by SLNB at 5- and 6-month follow-up, corresponding with a false-negative rate of 3.8%. Accordingly, in those with follow-up ≥ 24 months or false-negative SLNB within 24-month follow-up (*n* = 39), a sensitivity of 82% and a NPV of 93% was obtained. The false-negative rate of SLNB in this cohort without the use of the handheld gamma-camera would have been 7.6% (4/53), corresponding with a sensitivity of 64% and a NPV of 87%.

## Discussion

This study evaluated the use of the Crystal Cam handheld gamma-camera in 53 early-stage OSCC (cT1-3N0) patients undergoing SLNB. Overall, this handheld gamma-camera was able to preoperatively detect 95% of SLNs (116/122) as identified by conventional lymphoscintigraphy. When blinded for lymphoscintigraphy, 92% of SLNs were correctly identified by handheld gamma-camera. The marked location of SLNs by handheld gamma-camera and ^57^Co-penpoint marker was considered accurate in 98% of SLNs. Its complementary use during surgery led to the extirpation of additional ‘hot’ lymph nodes in 14 patients, which ultimately prevented two patients from otherwise being falsely staged negative for nodal disease (4%). The surgeon deemed the complementary use of the handheld gamma-camera helpful for intraoperatively localizing SLNs in 55% of patients.

When considering its accuracy in detecting and localizing SLNs, both preoperatively and intraoperatively, the clinical utility of the Crystal Cam handheld gamma-camera in early-stage OSCC appears similar to several other gamma-detecting imaging devices [12, 13, 17–21, 32]. This handheld gamma-camera proved to be particularly helpful when scanning for residual activity after harvesting a presumed SLN [19, 32]; most additional ‘hot’ lymph nodes (possibly remaining SLNs) were harvested in this manner. Furthermore, in several patients, tracking SLNs under conventional gamma-probe guidance was challenging and time-consuming. In these circumstances, the handheld gamma-camera provided more precise information on the SLN’s position, thus facilitating its localization and extirpation (surgeon score 3). The handheld gamma-camera was even considered helpful in 4 out of the 10 patients in whom fluorescence guidance was also available; its use resulted in the extirpation of 2 additional ‘hot’ lymph nodes in these patients. More efficient SLN localization may decrease the extent of exploration (with its associated postoperative fibrosis and risk of complications) required to harvest SLNs, which benefits an eventual complementary neck dissection, and may reduce the duration of surgery [12, 13, 20]. However, if more ‘hot’ lymph nodes are found and harvested on account of real-time radioguided imaging, the procedure may as well be prolonged [13].

In addition, the dual-isotope capability of this handheld gamma-camera has shown to enable accurate preoperative localization of designated SLNs by ^57^Co-penpoint marker. Owing to the close proximity of the handheld gamma-camera to the skin, the majority of SLNs can easily be localized and marked by the nuclear physician using this dual-isotope feature. Since the location of SLNs in definite surgical position may slightly differ from the preoperatively marked location, the handheld gamma-camera can also be used to adjust skin marks intraoperatively [12, 13]. Moreover, as the ^57^Co-penpoint marker is small and can easily be incorporated in a sterile setting using a surgical glove, the dual-isotope feature can even be used after the incision is made to further narrow down the exact location of SLNs. Accordingly, this handheld gamma-camera has been introduced within the clinical care of this institution.

There are a few shortcomings of the handheld gamma-camera used in this study. First, since it only provides a two-dimensional view, no real-time information on the depth of SLNs can be obtained. Second, as demonstrated by the overlooked and undetected SLNs using the handheld gamma-camera in this study, (handheld) gamma-cameras remain susceptible to the shine-through phenomenon and may, therefore, experience difficulties in detecting SLNs located near the injection site [9, 10, 12]. In addition, detecting SLNs with low radioactive uptake may be challenging. Especially in these situations, near-infrared fluorescence imaging can be of added value [12, 14]. Finally, one should be aware of intense radioactive signals originating from the contralateral neck when using the handheld gamma-camera, as these can be mistaken for a hotspot in the side of the neck being scanned. This issue can easily be overcome though by adjusting the view angle of the handheld gamma-camera relative to the hotspot.

Certain limitations of this study have to be acknowledged. Most importantly, since the handheld gamma-camera was used in all patients, several of its potential benefits (e.g., reduced duration of surgery, lower complication rate) cannot be assessed. Obviously, the surgical procedure cannot be performed twice in each patient and considerable interpatient variability renders randomization an unrealistic option. Therefore, determining its additional value on these matters is unfeasible. Second, due to the number of surgeons (n = 7) having used the handheld gamma-camera intraoperatively, examination bias is inevitable, even though handling this handheld gamma-camera is intuitive and interpretation of its images fairly straightforward [24]. Finally, the variability in radiotracers and radioactive dosage administered as well as protocols used for SLNB (i.e., single-day protocol, 2-day protocol) may have affected this study’s outcomes.

In conclusion, the results of this study demonstrate that the relatively inexpensive and portable Crystal Cam handheld gamma-camera offers reliable preoperative and intraoperative SLN localization in early-stage OSCC patients, which facilitates SLNB and might even reduce the risk of missing a malignant SLN during surgery. Still, detecting SLNs close to the injection site or with low radioactive uptake by handheld gamma-camera can be challenging. In those situations, complementary near-infrared fluorescence imaging may be of additional value.

## Data Availability

Data are available on a reasonable request.
